# Benign Pilomatricoma With Osseous Metaplasia: A Rare Case

**DOI:** 10.7759/cureus.30661

**Published:** 2022-10-25

**Authors:** Natasha Bhatti, Jai Ramchandani, Suzannah August, Willie Chong

**Affiliations:** 1 Dermatology, King's College London, London, GBR; 2 Anatomy, King's College London, London, GBR; 3 Dermatology, Poole General Hospital, Poole, GBR

**Keywords:** scalp lump, pilomatrixoma, benign adnexal tumours, calcifying epithelioma of malherbe, osseous metaplasia, pilomatricoma

## Abstract

Pilomatricomas are frequently misdiagnosed benign neoplasms of the skin derived from hair matrix cells. Pilomatricomas may undergo calcification and ossification although the latter is rare and poorly documented, with only eight cases reported since 2006. We present a case of pilomatricoma with osseous metaplasia arising from the scalp in an 87-year-old female patient. She was referred by her general practitioner via the two-week cancer referral pathway, for a suspicious lesion. On examination, there was a 2 x 1cm nodule, with protruding hardened yellow material, on the right side of the patient’s occipital scalp. The lump was hard, non-tender and had been present for 17 years. The surrounding area was bleeding and slightly ulcerated. A clinical diagnosis of a ruptured epidermal cyst was made, and the patient was prepared for excision under local anaesthesia. The excised lesion of 23 x 18 x 10mm with 22 x 9mm of skin was sent for histology. This revealed a partially ulcerated dermal lesion composed of islands of keratin with ‘ghost cell’ outlines. Foreign body granulomas, transition to mature lamellar bone, and foci of calcification were noted. There were no definite populations of basaloid cells and features of malignancy were not seen. These findings are consistent with benign pilomatricoma with osseous metaplasia. The patient was discharged 4 weeks later with satisfactory wound healing. Differentiating this tumour from other commonly encountered benign masses remains a challenge, as seen in this case which was initially misdiagnosed as a ruptured epidermal cyst.

## Introduction

Pilomatricoma, previously known as calcifying epithelioma of Malherbe, is a frequently misdiagnosed benign neoplasm of the skin associated with hair follicles [1]. The prevalence of this condition is highest in children and adolescents, however, it is also seen in the elderly [2]. It occurs more commonly in women compared to men [3], and in the upper extremities, mainly the head and neck region [4].

Pilomatricomas were first described in 1880 by Malherbe and Chenantis and most frequently appear as asymptomatic, firm, solitary nodules [3,4]. The tent sign is pathognomonic for pilomatricoma and this is seen when the skin over the tumour is stretched [5]. They can undergo malignant transformation into pilomatrix carcinomas and as such identification of these tumours is important, however, due to their rarity and scarce documentation, this remains a difficult task [6]. Calcification and focal ossification can occur in pilomatricoma, however, true osseous metaplasia is rare [7]. We present a rare case of pilomatricoma with osseous metaplasia arising from the scalp in an elderly female patient.

## Case presentation

**Figure 1:**
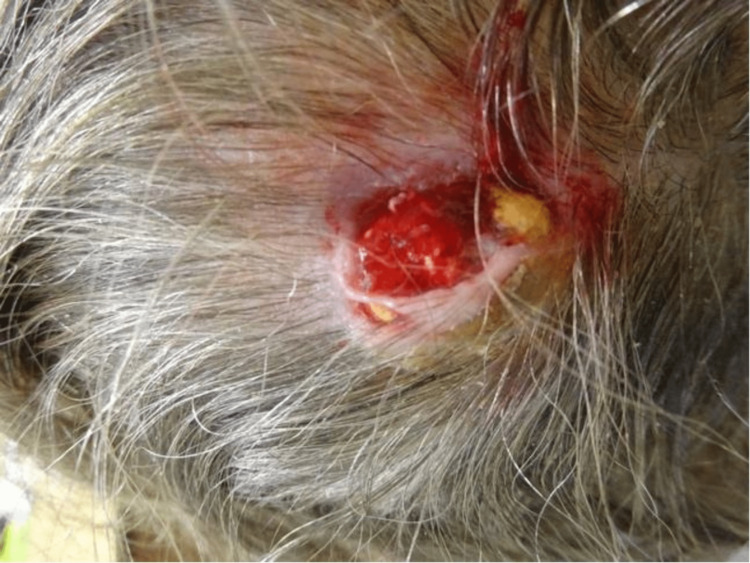
Patient presentation

An 87-year-old female patient was referred for a suspicious lesion by her general practitioner via the two-week cancer referral pathway. The patient presented to the dermatology department with a 17-year history of a lump that had recently become harder, on the right side of her scalp (Figure 1). The patient was otherwise fit and well and was not taking any regular medication. 

On examination, there was a 2 x 1cm nodule, with protruding hardened yellow material, on the right side of the patient’s occipital scalp. The lump was hard, non-tender and the surrounding area was bleeding and slightly ulcerated. A clinical diagnosis of a ruptured epidermal cyst was made, and the patient was prepared for excision under local anaesthesia. 

**Figure 2:**
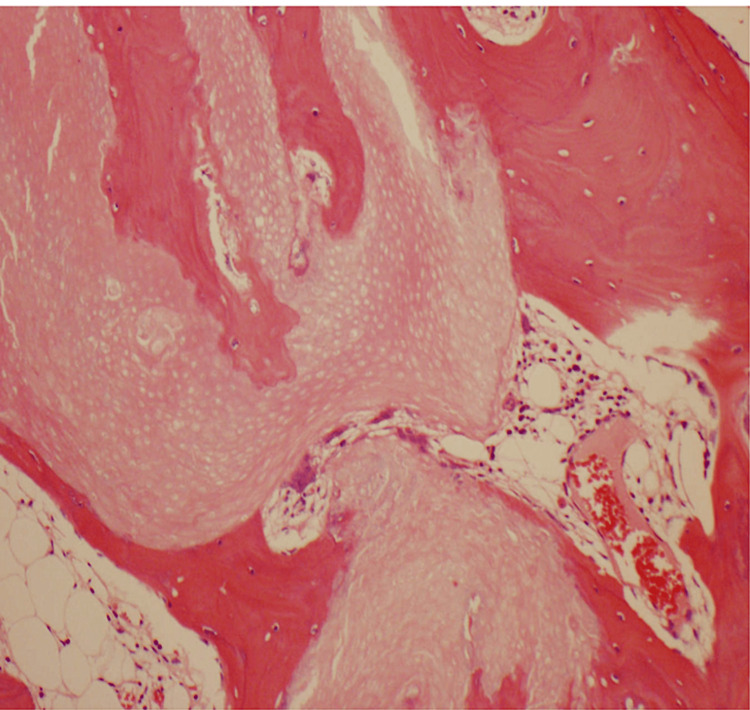
Hematoxylin and eosin stained section of the pilomatricoma highlighting the osseous metaplasia

The fully excised lesion of 23 x 18 x 10mm with 22 x 9mm of skin was sent for histology. This revealed a partially ulcerated dermal lesion composed of islands of keratin with ‘ghost cell’ outlines. Foreign body granulomas, transition to the mature lamellar bone, and foci of calcification were noted. There were no definite populations of basaloid cells and features of malignancy were not seen. These findings are consistent with benign pilomatricoma with osseous metaplasia (Figure 2) [8].

## Discussion

Pilomatricomas are rare skin neoplasms arising from the cortex of hair cells. Patients will often present as asymptomatic with a slow-growing, firm, non-tender, single nodule. The overlying skin is usually of normal colour and texture and may be visible as the tent sign, attributed to the attachment of the tumour to the epidermis [9]. Lesions are treated with excision under local anaesthesia and sent for histopathology to confirm the diagnosis. These tumours are usually benign, and recurrence is rare after complete resection. If there is a recurrence, malignant transformation should be considered. 

The tumour size ranges from 0.5 to 3cm in diameter [10], and is divided into four morphological subcategories; early stage, fully developed stage, early regressive stage, and late regressive stage. As the tumour progresses through the stages, basophilic cells within the lobules of the pilomatricoma lose their nuclei and increase their cytoplasm, transforming into eosinophilic shadow cells [5]. These cells can commonly undergo calcification (incidence of 69-89%), leading to hardening of the lesion, and also less commonly, ossification (incidence 15%) [11].

The pathogenesis of osseous metaplasia is unknown, although it is thought that bone morphogenic protein (BMP-2) mediates fibroblast metaplasia to form osteoblasts in late-stage pilomatricomas [1]. Pilomatricomas are clinically similar with or without osseous metaplasia, however, the former are firmer on palpation and have a long history of presentation. Shadow cells are observed in the histopathology of ossifying pilomatricoma [12]. The lack of basaloid cells in this case indicates a long-standing pilomatricoma with osseous metaplasia. 

Differentiating this tumour from other commonly encountered benign masses remains a challenge, as seen in this case which was initially misdiagnosed as a ruptured epidermal cyst. The diffuse yellow colour of the tumour seen in this case is not common in pilomatricomas. Characteristic red-blue discolouration over the lesion, associated with haemorrhage of the pilomatricomas [10], was not present. 

In February 2020, Bharti et al. [7] performed a literature review and identified eight cases of ossifying pilomatricoma between 2006 and 2020. The review revealed that 75% of cases occurred in females and no recurrence was recorded in any of the patients. This highlights the rare and benign nature of this condition. It is important to document these cases to raise awareness of pilomatricomas with rare histopathological findings, to ensure a correct diagnosis is reached and patient management is optimised. 

## Conclusions

Pilomatricoma with osseous metaplasia is rare and should be considered as a differential diagnosis in patients presenting with circumscribed, firm, non-tender nodules. The condition is often misdiagnosed, and correct diagnosis is often only established after excision and histological examination. 
